# Supplementary material for: Acceptance and compliance with micronutrient powder (MNP) among children aged 6–23 months in northern Nigeria
Source: PLOS Glob Public Health. 2022 Oct 17;2(10):e0000961. doi: 10.1371/journal.pgph.0000961 (PMC10022258; doi:10.1371/journal.pgph.0000961)
Supplement: S1 Dataset — (ZIP) [file pgph.0000961.s008.zip › S8_Dataset/Direct Observations.zip!/Direct Observations/12072015_A_GU_AS_2_DO.pdf]

1.1.1 Instrument F –Direct Observation Form

<<Instrument F Direct Observation Form>>
**PI Name:** PragyaMathema,Stephen Kodish<br>**Study Title:** Formative research to develop an infant and young child feeding (IYCF) intervention with micronutrient powder (MNP) for children aged 6 – 23 months<br>**PI Version/Date:** Version1.0/January 24, 2015

Date: _July 12, 2015____________Location: _purokayo community, LGA -Guyuk

Data Collector: Antiya Sanda Participant ID: A house near a bar

Main goals of this direct observation are to answer the following questions:

1. In this household, what would be the best way(s) to give nutritional supplements to young children? Why?

2. In this household, describe the food sharing that you observe throughout the meal. Are there any predictable patterns?

3. In this household, who is favored and how is this favoritism expressed during meal preparation and eating?

(TEAR OFF AT PERFORATED DOTTED LINE BELOW)

PARTICIPANT ID: __

Household Composition Table (Fill out as you do data collection)

INITIALS	SEX (M/F)	AGE (estimated)	Relationship to household	Description
AH	F	26 YRS	Mother	A woman with average weight
OM	M	60 Yrs	Father in law	An old man in his late 60's
TB	M	8 Yrs	Relation	A teenage boy
BG	F	5 Yrs	Relation	A black girl
YW	F	30 Yrs	Relation	A young woman wearing a black cloth
RT	F	13 Yrs	Younger sister	A teenage girl wearing a red dress
TB	M	25 yrs	Relation	A tall black man with no shirt on
SM	M	19 month	Son	A small boy
SB	F	45 years	Mother in law	A skinny black woman

PARTICIPANT ID: __________

TIME	ACTOR INITIALS	RECIPIENT INITIALS	WHAT HAPPENED	FOOD	Reactivity
8.00	Ah		Sweeping the house and corridor		
8:05	Ah		sweeping		
8:10	Ah		Sweeping the house		
8:15	Ah		Sweeping		
8:20	Ah		Sweeping		
8:25	Ah		Sweeping		
8:30	Ah		Sweeping		
8:35	Ah		Start fetching water from a big water pot		
8:40	Ah		fetching water from a big water pot		
8:45	Ah		Gather dirty plates		
8:50	Ah		Gather dirty plates		
8:55	Ah		Washing plates		
9:00	Ah		Washing the plates		
9:05	Ah		Washing the plates		
9:10	Ah		Washing the plates		
9:15	Ah		Washing the plates		
9:20	Ah		Rinsing the plates		
9:25	Ah		Rinsing the plates		
9:30	Ah		Rinsing the plates		
9:35	Ah		Washing cooking pots		
9:40	Ah		washing pots		

TIME	ACTOR INITIALS	RECIPIENT INITIALS	WHAT HAPPENED	FOOD	Reactivity
9:45	Ah		Washing pots with detergent and sand		
9:50	Ah		Rinsing pots		
9:55	Ah		Rinsing the pots		
10:00	Ah		Gather firewood		
10:05	Ah		Start making fire		
10:10	Ah		Making fire		
10:15	Ah		Pick a small pot and pour water into it	Water	
10:20	Ah		Covers the pot and put it on fire		
10:25	Ah		Bring a small bowl with guinea corn flour inside	Guinea corn	
10:30	Ah		Bring out sieve		
10:35	Ah		Sieving the guinea corn flour	Guinea corn	
10:40	Ah		Sieving the guinea corn flour	Guinea corn	
10:45	Ah		Sieving the guinea corn flour	Guinea corn	
10:50	Ah		Continues Sieving the guinea corn flour	Guinea corn	
10:55	Ah		Check water on fire	Water	
11:00	Ah		Poured little guinea corn flour into a bowl	Guinea corn	
11:05	Ah		Poured little water into it	Guinea corn and water	
11:10	Ah		Mixing of the flour and water	Guinea corn flour and water	
11:15	Ah		Opens the pot on fire		
11:20	Ah		Poured the mixture into the boiling water	Guinea corn and water	
11:25	Ah		Stirring to allow it to cook	Guinea corn flour	
11:30	Ah		Allow to cook	Guinea corn	
11:35	Ah		Remove pot from fire	Guinea	

TIME	ACTOR INITIALS	RECIPIENT INITIALS	WHAT HAPPENED	FOOD	Reactivity
				corn	
11:36	Ah		Poured the kunu into cup	Guinea corn	
11:37	Ah		Use spoon to stir the kunu	Guinea corn	
11:38	Ah		Still stirring with spoon to make it cold	Guinea corn	
11:39	Ah		Goes into the room to bring out MNP		
11:40	Ah		Unseal the MNP		
11:41	Ah		Pour MNP into cup of kunu	Guinea corn, MNP	
11:42	Ah		Stir with spoon to mix well	MNP, guinea corn	
11:43	Ah		Stirring with small spoon	MNP, guinea corn	
11:44	Ah		Taste the mixture with spoon	MNP, guinea corn	
11:45	Ah	Sm	Carry her child on the lap		
11:46	Ah	Sm	Start feeding the child with kunu mixed with MNP using a spoon and a cup	MNP, guinea corn	
11:47	Ah	Sm	feeding the child with kunu mixed with MNP using a spoon and a cup	MNP, guinea corn	
11:48	Ah	Sm	feeding the child with kunu mixed with MNP using a spoon and a cup	MNP, guinea corn	
11:49	Ah	Sm	feeding the child with kunu mixed with MNP using a spoon and a cup	MNP, guinea corn	
11:50	Ah	Sm	feeding the child with kunu mixed with MNP using a spoon and a cup	MNP, guinea corn	
11:51	Ah	Sm	Continue feeding the child with kunu mixed with MNP using a spoon and a cup	MNP, guinea corn	

TIME	ACTOR INITIALS	RECIPIENT INITIALS	WHAT HAPPENED	FOOD	Reactivity
11:52	Ah	Sm	Continue feeding the child with kunu mixed with MNP using a spoon and a cup	MNP, guinea corn	
11:53	Ah	Sm	Continue feeding the child with kunu mixed with MNP using a spoon and a cup	MNP, guinea corn	
11:54	Sm		Feed himself with cup alone		
11:55	Sm		Feeding himself with a small cup	MNP, Guinea corn	
11:56	Sm		Continues to feed himself with cup alone	MNP, Guinea corn	
11:57	Sm		Feeding himself with cup and playing at same time	MNP, Guinea corn	
11:59	Sm		Finish the kunu		
12:00	Sm		Drop cup on the ground		
12:05	Ah	Sm	Use water to clean his chest and body		
12:10	Ah	Sm	Clean the boy face with water		
12:15	Ah		Wipe with face with a small towel		
12:20	Ah	Sm	Clean his face with water		
12:25	Ah		Goes to the room and comes out with a shirt		
12:30	Ah	Sm	Start wearing shirt for the child		
12:35	Ah	Sm	Wearing a shirt for the child		
12:40	Ah		Pick the empty cup of kunu and spoon		
12:45	Ah		Start rinsing the cup and spoon with water		
12:55	Ah		Takes cup and spoon to the kitchen		
1:00	BG, Sm		Playing together		
1:05	BG, Sm		Playing together		

TIME	ACTOR INITIALS	RECIPIENT INITIALS	WHAT HAPPENED	FOOD	Reactivity
1:10	BG, Sm		Playing together		
1:15	Sm		Start sleeping		
1:20	Sm		Sleeping on the mat in the compound	Cerelac and water	
1:25	Sm		Sleeping		
1:30	Ah	Sm	Carry the child into the room and covered him with a wrapper		
1:35	Sm		Sleeping		
1:40	Sm		Sleeping		
1:45	Sm		Sleeping		
1:50	Sm		Sleeping		
1:55	Sm		Sleeping		
2:00	Sm		Sleeping		
2:05	Sm		Sleeping		
2:10	Sm		Sleeping		
2:15	Sm		Sleeping		
2:20	Sm		Sleeping		
2:25	Sm		Sleeping		
2:30	Sm		Sleeping		
2:35	Ah		Start gathering the fire wood again		
2:40	Ah		Start blowing the fire		
2:45	Ah		Fetches water in a pot and placed it on the fire		
2:50	Ah		Bring out a big bowl of guinea corn flour and sieve	Guinea corn flour	
2:55	Ah		Sieving the guinea corn	Guinea corn flour	
2:56	Ah		Sieving the guinea corn	Guinea corn flour	
2:57	Ah		Sieving the guinea corn	Guinea corn flour	
2:58	Ah		Sieving the guinea corn	Guinea corn flour	
2:59	Ah		Sieving the guinea corn	Guinea corn flour	
3:00	Ah		Sieving the guinea corn	Guinea	

TIME	ACTOR INITIALS	RECIPIENT INITIALS	WHAT HAPPENED	FOOD	Reactivity
				corn flour	
3:01	Ah		Sieving the guinea corn	Guinea corn flour	
3:02	Ah		Sieving the guinea corn	Guinea corn flour	
3:03	TB		Blowing fire		
3:04	TB		Fetches guinea corn flour and mix it water		
3:05	TB		Pour the mixture into the pot on fire		
3:06	TB		Stir with stick	Guinea corn	
3:07	TB		Removes the stick and cover the pot		
3:08	TB		Bring out different plates from the kitchen		
3:09	TB		Arrange the plates		
3:10	TB		Pick a bowl		
3:11	TB		Take a bowl of guinea corn	Guinea corn	
3:12	TB		Opens the pot on the fire		
3:13	TB		Add the guinea corn flour little by little		
3:14	TB		Uses a stick to stir the guinea corn flour and stir faster		
3:15	TB		Pour the remaining flour and stir faster	Guinea corn	
3:16	TB		Continues to stir the guinea corn flour		
3:17	TB		Stirring the guinea corn flour faster	Guinea corn flour	
3:18	TB		Removes the stick and covers the pot	Guinea corn	
3:19	TB		Allows the tuwo to cook	Guinea corn	
3:20	TB		Opens the pot and start serving tuwo	Guinea corn	
3:21	TB		Serving tuwo into different plates a		

TIME	ACTOR INITIALS	RECIPIENT INITIALS	WHAT HAPPENED	FOOD	Reactivity
3:23	TB		Serving tuwo	Guinea corn	
3:24	TB		Still serving tuwo	Guinea corn	
3:25	TB		Serving tuwo into 5 plates	Guinea corn	
3:26	TB		Serves all the tuwo and pour water into the pot		
3:27	TB		Bring down the pot from the fire		
3:28	TB		Start covering the plates with covers		
3:29	TB		Covering the plates		
3:30	TB		Start packing the plates to the kitchen		
3:31	TB		Packing all the plates to the kitchen		
3:32	Sm		Wakes up from sleep		
3:33	Sm		Uses a cup to fetch water from the pot	Water	
3:34	Sm		Drinking water	Water	
3:36	Sm		Drinking water	water	
3:37	Sm		Drinking water	water	
3:38	Sm		Drinking water	water	
3:39	TB		Bring an already cooked pot of soup from the kitchen	Okro soup	
3:40	TB		Place the pot of soup on the fire	Okro	
3:41	TB		Opens the pot of soup	Okro	
3:42	TB		Use spoon to stir the soup	Okro	
3:43			Stirring the soup		
3:44	TB		Taste soup with spoon	Okro	
3:45	TB		Tasting the soup	Okro	
3:46	TB		Taste the soup	Okro	
3:47	TB		Covers the pot of soup	Okro	
3:48	TB		Start serving the soup into small bowls	Okro soup	
3:49	TB		Serving soup	Okro soup	
3:50	TB		Serving soup		
3:51	TB		Covers the pot of soup and takes it to the room	Okro soup	
3:52	BG		Packing soup bowls	Okro	

TIME	ACTOR INITIALS	RECIPIENT INITIALS	WHAT HAPPENED	FOOD	Reactivity
				soup	
3:53	BG		Packing soup bowls		
3:54	BG		Carry food and soup to the mat		
3:55	Sm		Sit on the mat		
3:56	BG		Washing her hands with water		
3:57	BG	SM	Washing hand of the child with just water		
3:58	BG	Sm	Washing hands		
3:59	BG	Sm	Hand feeding the child with tuwo and okro soup	Okro soup, guinea corn	
4:00	BG		Feeding herself	Okro soup and guinea corn	
4:01	BG		Feeding		
4:02	BG	Sm	Continues hand feeding the chuld		
4:03	BG	Sm	Feeding the baby		
4:04	BG	Sm	Feeding the baby		
4:05	BG		Feeding herself		
4:06	BG		Feeding herself		
4:07	BG	Sm	Feeding the baby		
4:08	BG		Continues feeding herself		
4:09	BG	Sm	Gives the baby water to drink	Water	
4:10	BG	Sm	Gives the child water to drink	Water	
4:11	Sm		Drinking water	Water	

TIME	ACTOR INITIALS	RECIPIENT INITIALS	WHAT HAPPENED	FOOD	Reactivity
4:12	Sm		Drinking water		
4:13	BG	Sm	Continues feeding the child		
4:15	Sm		Playing alone		
4:20	BG		Continues feeding herself		
4:25	BG		Feeding herself	Okro, guinea corn	
4:30	BG		Feeding herself		
4:35	TB	Sm	Removing his cloth		
4:40	TB	Sm	Removing his cloth		
4:45	TB		Fetching water in a bucket		
4:50	TB		Fetching water		
4:55	TB	Sm	Start bathing her child with soap and water		
5:00	TB	Sm	Bathing her child		
5:05	TB	Sm	Bathing her child		
5:10	TB	Sm	Bathing her child		
5:15	TB	Sm	Use wrapper to clean the face of the child		
5:25	TB	Sm	Applying Vaseline on the body of the child		
5:30	TB	Sm	Rubbing Vaseline		
5:35	TB	Sm	Wears clothes for the child		
5:40	TB	Sm	Wears her child a shirt and a trouser		
5:45	TB	Sm	Applying baby powder on the child neck		
5:50	TB	Sm	Brush his hair with hair brush		
5:55	TB	Sm	Wear rubber sandal for the child		
6:00	TB	Sm	Carry the child on her arm		

PARTICIPANT ID: __ _______

Primary Questions (Complete after you complete data collection):

From what you have seen observing THIS HOUSEHOLD, please answer the following questions in detail:

Question	Write your detailed response here
1. In this household, what	The best way and time to give the MNP should be in the morning

would be the best way(s) to give nutritional supplements such as an MNP to young children to ensure compliance?<br><br>Why?	when child is hungry.<br><br>Because the semi solid foods are usually prepared for young children in the morning.
2. In this household, who is favored during meal preparation or feeding?<br><br>How is this favoritism expressed?	The young child is favoured during meal preparation and feeding.<br><br>The young child is served first before anyone else and fed by his cousin sister until his satisfy.
3. How could we promote the MNP supplement for this family that would attract their attention?<br><br>Who would they listen to?	The best way to promote the MNP would be through the mother and the cousin sister since they both take part in the feeding of the child.<br><br>They would listen to the mother and the cousin sister because they are always around and took part in the feeding practices and meal preparation for the entire family.
4. In this household, what would be the best ways to promote the MNP but limit how much it would be shared?<br><br>Why?	The best way to promote the MNP would be through the mother but the tendency that the MNP could be shared is very high because many people are living in this household.<br><br>The relations that live in this household which include young and old could use the MNP for other children or mishandled the product.

5. What other issues and concerns did you see with this family that are important for us to consider with regard to introduction of an MNP?<br><br>Why?	There no concern or issues in this household. However, when I was counting the remaining MNP I saw one was forcefully torn with teeth and it was left opened.<br><br>This could be that other children in the household tried to taste the product and realized it was tasteless and left it opened.<br>This shows that the mother did not properly keep the product out of reach of children.
6. Based on your experience observing this child today, do you believe reactivity negatively impacted your ability to see the food-related behaviors of the child?<br><br>Please explain your answer in detail.	Reactivity did not affect my stay and observation process.<br>They are use to having me around.<br><br>There was no such thing like reactivity today. Because all the members of this household are already used to having me around all day.
